# Lower starting dose of afatinib for the treatment of metastatic lung adenocarcinoma harboring exon 21 and exon 19 mutations

**DOI:** 10.1186/s12885-021-08235-3

**Published:** 2021-05-03

**Authors:** Yi-Chieh Chen, Ming-Ju Tsai, Mei-Hsuan Lee, Chia-Yu Kuo, Mei-Chiou Shen, Ying-Ming Tsai, Huang-Chi Chen, Jen-Yu Hung, Ming-Shyan Huang, Inn-Wen Chong, Chih-Jen Yang

**Affiliations:** 1 Department of Pharmacy, Kaohsiung Municipal Ta-Tung Hospital, Kaohsiung Medical University, Kaohsiung, Taiwan; 2 Graduate Institute of Medicine, College of Medicine, Kaohsiung Medical University, Kaohsiung, Taiwan; 3 Division of Pulmonary and Critical Care Medicine, Kaohsiung Medical University Hospital, Kaohsiung Medical University, Kaohsiung, Taiwan; 4 Faculty of Medicine, Kaohsiung Medical University, Kaohsiung, Taiwan; 5 Department of Internal Medicine, Kaohsiung Municipal Siaogang Hospital, Kaohsiung Medical University, Kaohsiung, Taiwan; 6 Department of Pharmacy, Kaohsiung Medical University Hospital, Kaohsiung Medical University, Kaohsiung, Taiwan; 7 Department of Internal Medicine, E-DA Cancer Hospital, Kaohsiung, Taiwan; 8 Respiratory therapy, College of Medicine, Kaohsiung Medical University, Kaohsiung, Taiwan; 9 Department of Internal Medicine, Kaohsiung Medical University Hospital, No. 100, Tzyou First Road, Kaohsiung City, Taiwan

**Keywords:** Epidermal growth factor receptor tyrosine kinase inhibitor, Afatinib, Lower starting dose, Adverse drug reaction

## Abstract

**Background:**

Afatinib has shown favorable response rates (RRs) and longer progression free survival (PFS) in lung cancer patients harboring EGFR mutations compared with standard platinum-based chemotherapy. However, serious adverse drug reactions (ADRs) limit the clinical application of afatinib.

**Methods:**

We designed a retrospective study, enrolling all patients with metastatic lung adenocarcinoma who were diagnosed and treated with 30 or 40 mg daily afatinib as their initial treatment in three Kaohsiung Medical University-affiliated hospitals in Taiwan.

**Results:**

A total of 179 patients were enrolled in the study, of which 102 (57%) and 77 (43%) received 30 mg and 40 mg afatinib daily as their initial treatment, respectively. The patients initially using 30 mg afatinib daily had a similar RR (75% vs. 83%, *p* = 0.1672), median PFS (14.5 vs. 14.8 months, log-rank *p* = 0.4649), and median OS (34.0 vs. 25.2 months, log-rank *p* = 0.5982) compared with those initially using 40 mg afatinib daily. Patients initially receiving 30 mg afatinib daily had fewer ADRs compared with those using 40 mg daily. The overall incidence of moderate and severe ADRs was significantly lower in patients receiving 30 mg afatinib daily compared with those using 40 mg daily (49% vs. 77%, *p = 0.002*); similar findings was observed in terms of severe ADRs (7% vs. 24%, *p < 0.0001*).

**Conclusion:**

Patients receiving 30 mg afatinib daily as their initial treatment had similar RR, PFS, OS, but significantly fewer serious ADRs, as compared with those using 40 mg as their starting dose.

## Highlights

1. The patients initially using 30 mg afatinib daily had a similar RR (75% vs. 83%, *p* = 0.1672), median PFS (14.5 vs. 14.8 months, log-rank *p* = 0.4649), and median OS (34.0 vs. 25.2 months, log-rank *p* = 0.5982) compared with those initially using 40 mg afatinib daily.
2. Patients with a lower starting dose had fewer ADRs including diarrhea, stomatitis, dry skin, acne and/or skin rash, and pruritis compared with patients receiving 40 mg as their starting dose. The overall incidence of grade 3 ADRs was significantly lower in patients receiving 30 mg afatinib daily compared with those receiving 40 mg (7% vs 24%, *p* < 0.0001).
3. Serious adverse drug reactions (ADRs) may limit the clinical application of a higher dose of afatinib (40 mg daily) because about 40% of them have to discontinue their treatment or reduce the dosage due to severe ADRs.

## Background

Lung cancer is a leading cause of cancer related mortality worldwide, including in Taiwan. Most lung cancer patients are diagnosed at an advanced stage meaning salvage therapy is recommended [1]. Platinum-based chemotherapy is a standard therapy for advanced stage lung cancer but has only been proven to have modest clinical efficacy [2, 3]; the response rates (RRs) to 1st line cytotoxic chemotherapy for advanced non-small cell lung cancer (NSCLC) are 30 to 40%, and all patients eventually develop resistance with a median survival of only 8 to 10 months [3].

In addition, chemotherapy causes a number of severe adverse drug reactions (ADRs), such as nausea, vomiting, hematological toxicity and some unexpected life-threatening complications [3, 4], which can cause poor quality of life. Therefore, many new treatment strategies have been developed to improve the clinical efficacy of chemotherapy and to lower its toxicity. Driver mutations are believed to be involved in cancer pathogenesis and small molecular drugs designed to target the signal transduction pathway can result in cell apoptosis or death; these are often accompanied by fewer ADRs than standard chemotherapy [5, 6].

Therefore, many new target therapies were developed which have been proven to have better clinical efficacy compared with standard platinum-based chemotherapy [5]. Several large-scale phase 3 clinical trials have shown that lung cancer patients harboring susceptible epidermal growth factor receptor (EGFR) mutations who received an EGFR tyrosine kinase inhibitor (TKI) have better clinical efficacy compared with platinum-based chemotherapy, in terms of overall RRs, progression free survival (PFS) and quality of life [7–11]. Afatinib is an irreversible, second-generation EGFR TKI [12] which has been shown to have better RR, PFS and overall survival (OS) when used in patients of lung cancer harboring susceptible EGFR mutations, compared with platinum-based chemotherapy [13, 14]. Furthermore, afatinib has been shown to have a significantly longer PFS and time-to-treatment failure compared with gefitinib when used as the initial *EGFR* TKI in a head-to-head phase 2B clinical trial;. Afatinib also has been proven to have significantly longer OS in patients of lung cancer with exon 19 deletions [15]. Therefore, afatinib is a promising EGFR TKI for the management of patients with lung cancer with EGFR mutations.

However, ADRs were reported in 11% of patients taking 40 mg afatinib daily and 4% of patients taking gefitinib [16]. A meta-analysis showed that in patients who received first- and second-generation EGFR TKIs, 40% experienced grade 3–4 ADRs, while the risk of grade 3–4 ADRs was lower for gefitinib (29.1%) than for erlotinib (54.1%) or afatinib (42.1%) [6] . Another pooled safety study concluded that grade 3–4 skin rash and diarrhea occurred significantly more frequently with afatinib therapy compared with erlotinib or gefitinib therapy [17]. Patients receiving afatinib treatment always have more ADRs compared with 1st generation EGFR TKIs in patients with EGFR mutations, and 28 to 53.3% of patients receiving standard 40 mg afatinib daily had to discontinue or reduce their dose due to severe ADRs in the phase 3 LUX-Lung 3 and LUX-Lung 6 trials [13, 14, 18]. Real-world data of 1st-line afatinib treatment showed that dose reduction occurred in 47.5 to 76.3% of cases [19, 20]. Dose reductions were mainly due to ADRs and were more common in females, East-Asian individuals and those with a lower body weight [21]. Therefore, methods for ameliorating ADRs whilst maintaining clinical efficacy are urgently needed for lung cancer patients receiving afatinib as their first-line therapy. A lower starting dose of afatinib was tested by clinicians in clinical practice and several trials [22]. In May 2014, the Taiwan Nation Health Insurance Bureau permitted both 30 mg and 40 mg afatinib daily as a first-line therapy for advanced lung adenocarcinoma with susceptible EGFR mutations.

Our preliminary report, a very small-scale study that only enrolled 48 patients with different starting doses, showed that patients who received 30 mg afatinib daily as the starting dose had non-inferior PFS with fewer severe ADRs [23]. We believe that fewer adverse events, especially fewer severe ADRs will result in good drug compliance and a better quality of life during lung cancer treatment.

Herein, we designed a larger-scale retrospective study to investigate whether patients of lung adenocarcinoma with susceptible EGFR mutations receiving a lower starting doses of afatinib had a similar clinical effectiveness and fewer severe ADRs compared with those taking a higher starting dose of afatinib in Taiwan.

## Methods

### Patient identification

Patients with metastatic lung adenocarcinoma who were diagnosed and treated between May 1st 2014 and July 31st 2019 in Kaohsiung Medical University Hospital (KMUH), Kaohsiung Municipal Ta-Tung Hospital and Kaohsiung Municipal Siaogang Hospital (all Kaohsiung Medical University-affiliated hospitals) in Taiwan, were identified and followed until Dec 31st, 2019. The diagnosis of lung adenocarcinoma was confirmed pathologically according to the World Health Organization pathology classification. Tumor staging was assessed according to the Seventh American Joint Committee Cancer Staging System and confirmed by a multidisciplinary lung cancer team. All adenocarcinoma specimens were analyzed using an EGFR RGQ kit (Qiagen, UK). The protocol was developed and validated by the Division of Molecular Diagnostics, Department of Laboratory Medicine, KMUH, and utilized amplification refractory mutation specific (ARMS) polymerase chain reactions (PCRs) and Scorpion technologies for detection; direct sequencing was performed if a negative result was found in the ARMS PCR. The examination techniques were consistent with our previous studies [23–27].

In the current study, we enrolled all individuals with exon 19 deletions and exon 21 L858R point mutations, and excluded those with resistant mutation; they were all treatment-naïve and were treated with either 30 or 40 mg afatinib daily as their first-line treatment for stage IV metastatic lung adenocarcinoma. Baseline clinical characteristics were determined by retrospective chart review, including age at diagnosis, sex, weight, height, Eastern Cooperative Oncology Group (ECOG) performance status, glomerular filtration rate, smoking history, hepatitis B, hepatitis C, tuberculosis history, family history, thyroid transcription factor-1 (TTF-1) stain, programmed death-ligand 1 (PDL-1) stain, EGFR mutation, TNM status, and number of metastatic sites/organs on initial diagnosis.

The initial treatment response was classified based on serial imaging studies using the revised Response Evaluation Criteria in Solid Tumors (RECIST 1.1) criteria. The PFS and OS of the first-line afatinib treatment were defined as the time from the start of the first treatment to the date of disease progression on an imaging examination, and the date of death, respectively. ADRs were recorded by physicians and graded according to the Common Terminology Criteria for Adverse Events version 4.0.

### Statistical analysis

Categorical variables and continuous variables were compared using χ^2^ test and Student’s t-test, respectively. Survival times were estimated using the Kaplan-Meier method, with differences between the two groups compared using the log-rank test. Univariate Cox regression analyses were performed to identify the factors associated with PFS and OS. Using a backward variable selection method, keeping only variables with *p* values < 0.1, we developed reduced multivariable models with Cox regression analyses to determine independent predictive factors for PFS and OS. Hazard ratios (HR) with 95% confidence intervals (CIs) of the factors are reported. All statistical analyses were performed using SAS software (version 9.4 for Windows, SAS Institute Inc., Cary, NC, USA). Statistical significance was set at a two-sided *p* value of < 0.05.

## Results

During the study period, a total of 179 patients with stage IV lung adenocarcinoma harboring exon 19 deletions or an exon 21 *L858R* point mutation who received afatinib as their first-line therapy were enrolled (Table 1). Of these patients, 102 (57%) received 30 mg afatinib daily and 77 (43%) received 40 mg daily as their initial treatment. Patients receiving 30 mg daily as their initial dose, compared with those receiving 40 mg afatinib daily, were significantly older (65.7 ± 9.3 vs. 62.4 ± 9.3 years, *p* = 0.0199), had a significantly lower weight (58.2 ± 12.5 vs. 62.4 ± 9.9 kg, *p* = 0.014) and a significantly lower body surface area (1.6 ± 0.2 vs. 1.7 ± 0.2 m^2^ *p* = 0.0078), and were more likely to be female (72% vs. 48%). Patients in both groups has similar number of metastatic sites at the initial diagnosis (*p* = 0.2360), while significantly more patients with brain metastasis received 40 mg daily of afatinib as their initial dose, rather than 30 mg daily (42% vs. 21%, *p* = 0.0023). There was no significant difference in terms of other metastatic sites. There were no significant differences in body height, smoking history, glomerular filtration rate, serum albumin level, serum levels of liver enzymes, tuberculosis history, family history, performance status, TTF-1 staining, PDL-1 staining, and the EGFR gene mutation site (exon 19 or 21) of the cancer specimens between the two groups.

**Table 1:** Clinical characteristics and treatment responses for all patients

Variables	All patients	Afatinib 30 mg daily	Afatinib 40 mg daily	***P*** value
N	179	102	77	
Age (year)	64.3 ± 9.4	65.7 ± 9.3	62.4 ± 9.3	0.0199
Age group:				0.0212
< 65 years old	80 (45%)	38 (37%)	42 (55%)	
≥ 65 years old	99 (55%)	64 (63%)	35 (45%)	
Sex				0.0014
Female	110 (61%)	73 (72%)	37 (48%)	
Male	69 (39%)	29 (28%)	40 (52%)	
Smoking history:				0.9349
Never smoker	140 (78%)	80 (78%)	60 (78%)	
Ever smoker	39 (22%)	22 (22%)	17 (22%)	
Height (cm)	160 ± 8	159.1 ± 7.7	161.2 ± 8.4	0.0905
Weight (kg)	60 ± 11.6	58.2 ± 12.5	62.4 ± 9.9	0.0140
Body mass index (kg/m^2^)	23.3 ± 3.6	22.8 ± 3.7	24 ± 3.4	0.0339
Body surface area (m^2^)	1.6 ± 0.2	1.6 ± 0.2	1.7 ± 0.2	0.0078
Serum creatinine level	0.8 ± 0.3	0.8 ± 0.3	0.8 ± 0.3	0.4561
eCCr-CG (mL/min) ^a^	77.1 ± 26.8	73.7 ± 25.9	81.5 ± 27.5	0.0566
eGFR-MDRD (mL/min/1.73m^2^) ^b^	88.8 ± 27	88 ± 26.8	89.8 ± 27.4	0.6628
Serum albumin (mg/dL)	4.0 ± 0.5	4.0 ± 0.4	4.1 ± 0.5	0.2751
Serum glutamic oxaloacetic transaminase (U/L)	29.6 ± 22.8	31.4 ± 28.7	27.3 ± 10.4	0.1835
Serum glutamic pyruvic transaminase (U/L)	26.5 ± 25.1	26.7 ± 28.5	26.3 ± 20	0.9178
Hepatitis B: ^c^				0.4999
Negative	147 (84%)	86 (85%)	61 (81%)	
Positive	29 (16%)	15 (15%)	14 (19%)	
Hepatitis C: ^c^				0.0370
Negative	165 (94%)	98 (97%)	67 (89%)	
Positive	11 (6%)	3 (3%)	8 (11%)	
Old tuberculosis:				0.8901
Negative	174 (97%)	99 (97%)	75 (97%)	
Positive	5 (3%)	3 (3%)	2 (3%)	
Family history:				0.0859
Negative	168 (94%)	93 (91%)	75 (97%)	
Positive	11 (6%)	9 (9%)	2 (3%)	
Performance status while starting afatinib:				0.1024
ECOG ≤1	146 (82%)	79 (77%)	67 (87%)	
ECOG ≥2	33 (18%)	23 (23%)	10 (13%)	
TTF-1 stain: ^c^				0.8901
Positive	170 (100%)	99 (100%)	71 (100%)	
PDL-1 stain: ^c^				0.4732
Absence	33 (38%)	16 (34%)	17 (41%)	
Presence	55 (63%)	31 (66%)	24 (59%)	
*EGFR* gene mutation site: ^d^				
Exon 19	95 (53%)	60 (59%)	35 (45%)	0.0760
Exon 21	85 (47%)	42 (41%)	43 (56%)	0.0517
TNM staging:				
N2–3	112 (63%)	67 (66%)	45 (58%)	0.3214
M1a-c	179 (100%)	102 (100%)	77 (100%)	0.5136
Number of metastatic sites/organs (1–2 vs. > 2)				0.2360
1 site	64 (36%)	41 (40%)	23 (30%)	
2 sites	69 (39%)	39 (38%)	30 (39%)	
≥ 3 sites	46 (26%)	22 (22%)	24 (31%)	
Metastatic site/organ on initial diagnosis				
Brain	53 (30%)	21 (21%)	32 (42%)	0.0023
Lung	79 (44%)	40 (39%)	39 (51%)	0.1272
Pleura (or with pleural effusion)	80 (45%)	47 (46%)	33 (43%)	0.6678
Bone	99 (55%)	59 (58%)	40 (52%)	0.4322
Liver	22 (12%)	15 (15%)	7 (9%)	0.2573
Adrenal	16 (9%)	8 (8%)	8 (10%)	0.5544
Other sites	11 (6%)	5 (5%)	6 (8%)	0.4253

Data are presented in mean ± standard deviation (SD) or n (%)

^a^*eCCr-CG* estimated creatinine clearance rate using Cockcroft-Gault equation, while CrCl was multiplied by 0.85 for female patients

^b^ *eGFR-MDRD* estimated glomerular filtration rate using Modification of Diet in Renal Disease equation. GFR was multiplied by 0.742 and 1.212 for female patients and African-American, respectively

^c^Including missing values

^d^A patient had EGFR mutation in both exon 19 and exon 21

### Outcomes for 30 mg and 40 mg afatinib daily as the first-line treatment

**Fig. 1:**
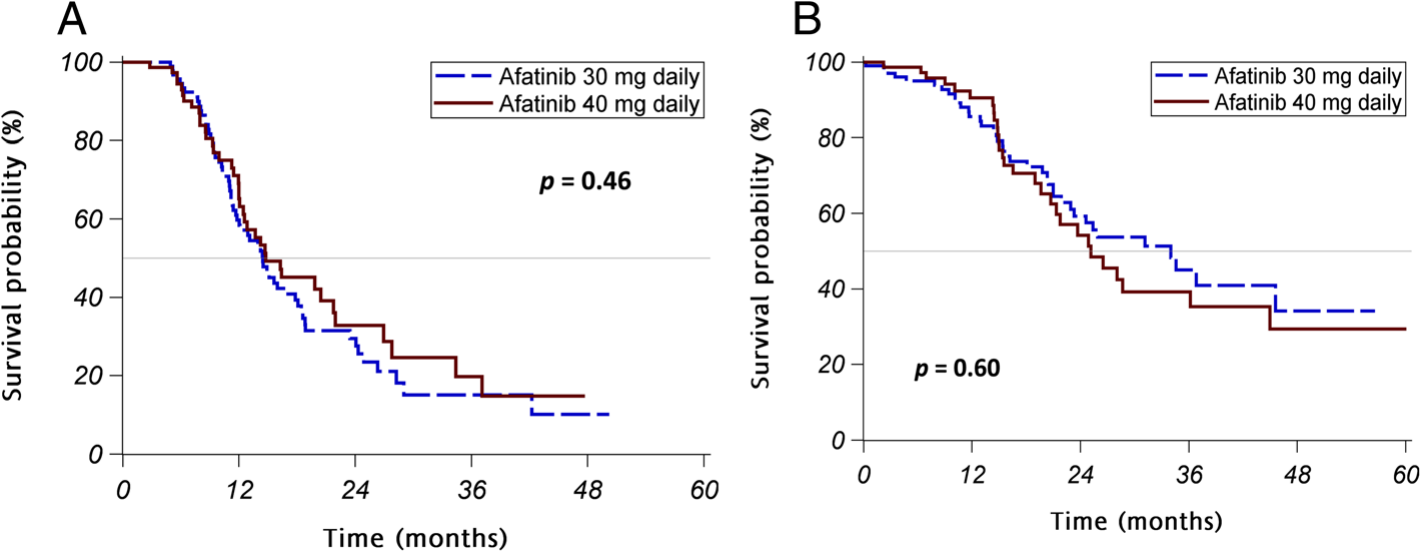
*PFS and OS for afatinib treatment.* **a** PFS in patients receiving different initial doses of afatinib. **b** OS for patients receiving different initial doses of afatinib

Patients initially receiving 30 mg afatinib daily had similar response rates (75% vs. 83%; *p* = 0.1672) and similar disease control rates (99% vs. 96%) compared with those initially receiving 40 mg afatinib daily (Table 2). The PFS was not significantly different between patients receiving 30 mg and 40 mg afatinib daily (median PFS: 14.5 vs. 14.8 months, log-rank *p* = 0.4649; Fig. 1a). In terms of OS, there was no significant difference between the two patient groups (median OS: 34.0 months vs. 25.2 months, log-rank *p* = 0.5982; Fig. 1b). Notable, only 13 patients (13%) receiving 30 mg afatinib and 5 patients (6%) receiving 40 mg afatinib daily as their initial treatment received osimertinib after developing acquired resistance to afatinib. Other patients chose to have platinum-based chemotherapy or hospice care as their second-line management.

**Table 2:** Initial treatment response to different initial afatinib doses

Variables	All patients	Afatinib 30 mg daily	Afatinib 40 mg daily	***P*** value
**Initial response to afatinib treatment -n (%)**				0.1661
Complete response	2 (1%)	1 (1%)	1 (1%)	
Partial response	138 (77%)	75 (74%)	63 (82%)	
Stable disease	35 (20%)	25 (25%)	10 (13%)	
Progressive disease	4 (2%)	1 (1%)	3 (4%)	
**Disease control rate with afatinib treatment (%)**	175 (98%)	101 (99%)	74 (96%)	0.1913
**Response rate with afatinib treatment (%)**	140 (78%)	76 (75%)	64 (83%)	0.1672

To identify factors associated with PFS, we built several Cox regression models (Table 3). In the univariate analyses, only the number of metastatic sites ≥3, pleural metastasis (or with pleural effusion), and bone metastasis were significantly risk factors for worse PFS, while dose reduction was associated with better PFS. In the model 1 of multivariable analysis, we found two independent predicting factors for PFS, higher number of metastatic sites (≥3) (HR [95% CI]: 1.83 [1.19–2.84], *p* = 0.0065) and dose reduction (HR [95% CI]: 0.57 [0.33–0.99], *p* = 0.0467). In the model 2 of multivariable analysis, considering the detailed metastatic sites instead of number of metastatic sites, we found exon 21 (HR [95% CI]: 1.56 [1.04–2.36], *p* = 0.0336), pleural metastasis/effusion (HR [95% CI]: 1.77 [1.17–2.69], *p* = 0.0075), and bone metastasis (HR [95% CI]: 1.69 [1.13–2.55], *p* = 0.0116) were independent risk factors for poorer PFS, while dose reduction showed a trend toward better PFS (HR [95% CI]: 0.59 [0.34–1.03], *p* = 0.0640).

**Table 3:** Cox regression analyses to identify the factors associated with progression-free survival (PFS)

Variables	Univariate analysis		Multivariable analysis - model 1 ^**a**^		Multivariable analysis - model 2 ^**a**^	
	HR [95% CI]	***P*** value	HR [95% CI]	***P*** value	HR [95% CI]	***P*** value
Afatinib dose (40 mg daily vs. 30 mg daily)	0.86 [0.58–1.29]	0.4657				
Sex (male vs. female)	1.19 [0.80–1.79]	0.3920				
Age (≥65 vs. < 65)	0.79 [0.53–1.17]	0.2333				
Smoking history (ever smokers vs. never smokers)	1.16 [0.71–1.89]	0.5659				
ECOG (≥2 vs. ≤1)	1.11 [0.67–1.84]	0.6762				
Exon 21 vs. exon 19 ^b^	1.31 [0.89–1.95]	0.1751			1.56 [1.04–2.36]	0.0336
Number of metastatic sites/organs (1–2 vs. ≥3)	1.86 [1.20–2.88]	0.0055	1.83 [1.19–2.84]	0.0065		
Metastatic site/organ on initial diagnosis: (yes vs. no)						
Brain	0.96 [0.62–1.49]	0.8479				
Lung	0.96 [0.64–1.44]	0.8318				
Pleura (or with pleural effusion)	1.57 [1.05–2.35]	0.0292			1.77 [1.17–2.69]	0.0075
Bone	1.52 [1.02–2.27]	0.0385			1.69 [1.13–2.55]	0.0116
Liver	1.50 [0.89–2.54]	0.1266				
Adrenal gland	0.96 [0.50–1.84]	0.8955				
Other site	0.84 [0.34–2.07]	0.7055				
Dose reduction (yes vs. no)	0.56 [0.32–0.98]	0.0407	0.57 [0.33–0.99]	0.0467	0.59 [0.34–1.03]	0.0640

^a^Multivariable Cox regression models were built using backward variable selection method, keeping only variables with *p* values less than 0.1. Number of metastatic sites/organs was considered while building model 1, whereas the detailed metastatic sites were considered while building model 2

^b^The patient with mutation in both exon 21 and exon 19 was arbitrary classified in to exon 21 group

We also built several Cox regression models (Table 4) to identify factors associated with OS. In the univariate analyses, male, smoking history, poorer performance status (ECOG ≥2) were significant risk factors associated with poorer OS. The model 1 of multivariable analysis showed that male (HR [95% CI]: 1.97 [1.21–3.22], *p* = 0.0066) and poorer performance status (HR [95% CI]: 2.28 [1.35–3.85], *p* = 0.0021) were independently associated with poorer OS, while the higher number of metastatic sites (≥3) was only associated with a trend toward poorer OS (HR [95% CI]: 1.61 [0.96–2.68], *p* = 0.0709). In the model 2 of multivariable analyses, we found three independent predicting risk factors for OS, including smoking history (HR [95% CI]: 2.37 [1.35–4.17], *p* = 0.0028), poorer performance status (HR [95% CI]: 3.00 [1.74–5.15], *p* < 0.0001), bone metastasis (HR [95% CI]: 1.73 [1.04–2.87], *p* = 0.0341), while adrenal gland metastasis was associated with better OS (HR [95% CI]: 0.33 [0.12–0.92], *p* = 0.0338).

**Table 4:** Cox regression analyses to identify the factors associated with overall survival (OS)

Variables	Univariate analysis		Multivariable analysis - model 1 ^**a**^		Multivariable analysis - model 2 ^**a**^	
	HR [95% CI]	***P*** value	HR [95% CI]	***P*** value	HR [95% CI]	***P*** value
Afatinib dose (40 mg daily vs. 30 mg daily)	1.14 [0.70–1.84]	0.5986				
Sex (male vs. female)	1.97 [1.22–3.20]	0.0058	1.97 [1.21–3.22]	0.0066		
Age (≥65 vs. < 65)	0.93 [0.58–1.51]	0.7729				
Smoking history (ever smokers vs. never smokers)	1.90 [1.10–3.29]	0.0210			2.37 [1.35–4.17]	0.0028
ECOG (≥2 vs. ≤1)	2.46 [1.46–4.15]	0.0007	2.28 [1.35–3.85]	0.0021	3.00 [1.74–5.15]	< 0.0001
Exon 21 vs. exon 19 ^b^	0.95 [0.59–1.54]	0.8334				
Number of metastatic sites/organs (1–2 vs. ≥3)	1.6 [0.96–2.67]	0.0690	1.61 [0.96–2.68]	0.0709		
Metastatic site/organ on initial diagnosis: (yes vs. no)						
Brain	1.54 [0.93–2.55]	0.0967				
Lung	1.08 [0.66–1.76]	0.7713				
Pleura (or with pleural effusion)	1.32 [0.82–2.12]	0.2584				
Bone	1.64 [0.99–2.70]	0.0532			1.73 [1.04–2.87]	0.0341
Liver	1.28 [0.68–2.39]	0.4395				
Adrenal gland	0.45 [0.16–1.25]	0.1264			0.33 [0.12–0.92]	0.0338
Other site	1.09 [0.40–3.01]	0.8668				
Dose reduction (yes vs. no)	1.08 [0.61–1.92]	0.7903				

^a^Multivariable Cox regression models were built using backward variable selection method, keeping only variables with *p* values less than 0.1. Number of metastatic sites/organs was considered while building model 1, whereas the detailed metastatic sites were considered while building model 2

^b^The patient with mutation in both exon 21 and exon 19 was arbitrary classified in to exon 21 group

### ADRs for patients using 30 mg or 40 mg afatinib daily as the first-line treatment

The most common ADRs in patients taking afatinib included acne and/or skin rash (81%), diarrhea (74%), dry skin (61%), and paronychia (51%) (Table 5). Patients receiving 30 mg afatinib daily had a lower incidence of diarrhea (68% vs. 82%), acne and/or skin rash (78% vs. 84%), dry skin (60% vs. 64%) and pruritis (23% vs 43%) compared with those receiving 40 mg afatinib daily. In terms of the maximal grade of ADRs, patients receiving 30 mg daily had less severe events than those receiving 40 mg daily (*p* < 0.0001). The patients receiving 30 mg afatinib daily initially, compared than those taking 40 mg afatinib daily, had a significantly lower overall incidence of moderate and severe (at least grade 2) (49% vs. 77%, *p* = 0.0002) and severe (at least grade 3) (7% vs. 24%, *p* < 0.0001) adverse drug reactions, particularly in diarrhea and adverse events involving skin. The incidences of drug-induced hepatitis and interstitial lung disease were very low and no significant differences were observed between the 30 mg and 40 mg groups in the present retrospective study.

**Table 5:** Adverse drug reactions related to different initial afatinib dosages

Adverse events	All patients	Afatinib 30 mg daily	Afatinib 40 mg daily	***P*** value
**Maximal grade of events**				< 0.0001
No	8 (4%)	2 (2%)	6 (8%)	
Grade 1	62 (35%)	50 (49%)	12 (16%)	
Grade 2	78 (44%)	43 (42%)	35 (45%)	
Grade 3	31 (17%)	7 (7%)	24 (31%)	
**Presence of any moderate and severe (≥ grade 2) adverse events**	109 (61%)	50 (49%)	59 (77%)	0.0002
**Details of moderate and severe (≥ grade 2) adverse events**				
Diarrhea	49 (27%)	13 (13%)	36 (47%)	< 0.0001
Stomatitis	14 (8%)	4 (4%)	10 (13%)	0.0253
Paronychia	50 (28%)	29 (28%)	21 (27%)	0.8642
Acne and/or skin rash	61 (34%)	17 (17%)	44 (57%)	< 0.0001
Dry skin	9 (5%)	1 (1%)	8 (10%)	0.0043
Pruritus	9 (5%)	1 (1%)	8 (10%)	0.0043
Adverse events involving skin ^a^	62 (35%)	18 (18%)	44 (57%)	< 0.0001
Hepatitis	4 (2%)	1 (1%)	3 (4%)	0.1913
Interstitial lung disease	0 (0%)	0 (0%)	0 (0%)	
**Presence of any severe (≥ grade 3) adverse events**	31 (17%)	7 (7%)	24 (31%)	< 0.0001
**Details of severe (≥ grade 3) adverse events**				
Diarrhea	14 (8%)	2 (2%)	12 (16%)	0.0008
Stomatitis	3 (2%)	2 (2%)	1 (1%)	0.7326
Paronychia	6 (3%)	2 (2%)	4 (5%)	0.2340
Acne and/or skin rash	11 (6%)	0 (0%)	11 (14%)	< 0.0001
Dry skin	0 (0%)	0 (0%)	0 (0%)	
Pruritus	1 (1%)	0 (0%)	1 (1%)	0.2484
Adverse events involving skin ^a^	11 (6%)	0 (0%)	11 (14%)	< 0.0001
Hepatitis	1 (1%)	1 (1%)	0 (0%)	0.3836
Interstitial lung disease	0 (0%)	0 (0%)	0 (0%)	
**Details of adverse events**				
Diarrhea				< 0.0001
No	47 (26%)	33 (32%)	14 (18%)	
Grade 1	83 (46%)	56 (55%)	27 (35%)	
Grade 2	35 (20%)	11 (11%)	24 (31%)	
Grade 3	14 (8%)	2 (2%)	12 (16%)	
Stomatitis				0.0214
No	125 (70%)	70 (69%)	55 (71%)	
Grade 1	40 (22%)	28 (27%)	12 (16%)	
Grade 2	11 (6%)	2 (2%)	9 (12%)	
Grade 3	3 (2%)	2 (2%)	1 (1%)	
Paronychia				0.6304
No	87 (49%)	49 (48%)	38 (49%)	
Grade 1	42 (23%)	24 (24%)	18 (23%)	
Grade 2	44 (25%)	27 (26%)	17 (22%)	
Grade 3	6 (3%)	2 (2%)	4 (5%)	
Acne and/or skin rash				< 0.0001
No	34 (19%)	22 (22%)	12 (16%)	
Grade 1	84 (47%)	63 (62%)	21 (27%)	
Grade 2	50 (28%)	17 (17%)	33 (43%)	
Grade 3	11 (6%)	0 (0%)	11 (14%)	
Dry skin				0.0171
No	69 (39%)	41 (40%)	28 (36%)	
Grade 1	101 (56%)	60 (59%)	41 (53%)	
Grade 2	9 (5%)	1 (1%)	8 (10%)	
Pruritus				0.0381
No	112 (63%)	68 (67%)	44 (57%)	
Grade 1	58 (32%)	33 (32%)	25 (32%)	
Grade 2	8 (4%)	1 (1%)	7 (9%)	
Grade 3	1 (1%)	0 (0%)	1 (1%)	
Adverse events involving skin ^a^				< 0.0001
No	24 (13%)	16 (16%)	8 (10%)	
Grade 1	93 (52%)	68 (67%)	25 (32%)	
Grade 2	51 (28%)	18 (18%)	33 (43%)	
Grade 3	11 (6%)	0 (0%)	11 (14%)	
Hepatitis				0.1382
No	172 (96%)	100 (98%)	72 (94%)	
Grade 1	3 (2%)	1 (1%)	2 (3%)	
Grade 2	3 (2%)	0 (0%)	3 (4%)	
Grade 3	1 (1%)	1 (1%)	0 (0%)	
Interstitial lung disease				0.2166
Grade 1	2 (1%)	2 (2%)	0 (0%)	

^a^Adverse events involving included acne, skin rash, dry skin, and pruritus

More patients receiving initial afatinib dose of 40 mg daily required dose reduction (or discontinuation) than those receiving 30 mg daily initially (40% vs. 8%, *p* < 0.0001) (Table 6).

**Table 6:** Category of afatinib dose reduction

Variables	All patients	Afatinib 30 mg daily	Afatinib 40 mg daily	***P*** value
**Dose reduction events**	39 (22%)	8 (8%)	31 (40%)	< 0.0001
**Category of afatinib dose reduction**				
No change	140 (78%)	94 (92%)	46 (60%)	
40 mg taper down to 30 mg	22 (12%)		22 (29%)	
40 mg taper down to 20 mg	7 (4%)		7 (9%)	
40 mg taper down to 0 mg	2 (1%)		2 (3%)	
30 mg taper down to 20 mg	1 (1%)	1 (1%)		
30 mg taper down to 15 mg	5 (3%)	5 (5%)		
30 mg taper down to 0 mg	2 (1%)	2 (2%)		

### Cancer recurrence in patients using 30 mg or 40 mg afatinib daily as a first-line treatment

The initial afatinib dose of 30 mg daily and 40 mg daily showed similar recurrence rate (54% vs. 45%, *p* = 0.2620) (Table 7). In terms of the recurrent sites, patients receiving 30 mg daily initially had a higher incidence of bone metastasis as the recurrent site compared with the 40 mg group (13% vs. 4%, *p* = 0.0399). Notably, there was no significant difference in the occurrence of central nervous system (including brain or leptomeningeal) metastasis as the recurrent site between patients in the 30 mg and 40 mg afatinib groups (18% vs. 21%, *p* = 0.5969).

**Table 7:** Sites of cancer recurrence in lung adenocarcinoma patients with different initial afatinib dosages

Recurrence	All patients	Afatinib 30 mg daily	Afatinib 40 mg daily	***P*** value
**Number of recurrent sites -n (%)**				0.3222
No recurrence	89 (50%)	47 (46%)	42 (55%)	
1 site	67 (37%)	43 (42%)	24 (31%)	
≥ 2 sites	23 (13%)	12 (12%)	11 (14%)	
**Details of recurrent sites**				
Central nervous system	34 (19%)	18 (18%)	16 (21%)	0.5969
Lung	38 (21%)	23 (23%)	15 (19%)	0.6192
Pleura or pleural effusion	16 (9%)	11 (11%)	5 (6%)	0.3191
Bone	16 (9%)	13 (13%)	3 (4%)	0.0399
Liver	8 (4%)	5 (5%)	3 (4%)	0.7471
Other sites	7 (4%)	2 (2%)	5 (6%)	0.1214

## Discussion

To the best of our knowledge, this retrospective trial is the largest evaluation of patients receiving 30 mg afatinib daily as the starting dose for the treatment of metastatic lung adenocarcinoma harboring exon 21 L858R point mutations or exon 19 deletions. We demonstrated that patients who received 30 mg afatinib daily as their starting dose had similar RRs, PFS, and OS compared with patients who received 40 mg daily as their starting dose, and they also had fewer severe ADRs.

The patients receiving 30 mg afatinib daily as the initial dose tended to be older, female sex, smaller in body size (less weight, lower body mass index, and lower body surface area), compared with those starting with 40 mg daily. These results are similar to previous studies [23, 28].

The patients initially using 30 mg afatinib daily had similar RRs as the 40 mg afatinib group, and the RR was comparable with previous studies (61–74%) [29]. In two phase 3 clinical trials, LUX-Lung 3 and LUX-Lung 6, the median PFS among patients of lung adenocarcinoma harboring EGFR mutations taking 40 mg afatinib as their initial dose was 10.9 and 13.6 months, respectively. A real-word practice study in Japan that enrolled 128 patients reported a median PFS of 17.8 months [20], while a phase 2 study which used a lower starting dose of 20 mg daily afatinib that increased in 10-mg increments up to 50 mg daily, reported a PFS of 15.2 months [22]. Another phase 2 study that enrolled 40 elderly patients had a shorter PFS of 12.9 months [19]. The current study revealed that the median PFS of patients of lung adenocarcinoma with exon 19 or exon 21 mutation taking afatinib 30 or 40 mg afatinib daily as their initial treatment was 14.5 and 14.8 months, respectively; no significant difference in the PFS was observed between the two groups, and the result was similar to our previous small-scaled study [23].

In terms of OS, the phase 3 LUX-Lung 3 and LUX-Lung 6 trials reported that the median OS was 31.4 and 33.3 months, respectively. Tanaka et al. performed a real-world study of first-line afatinib in Japan, showing a median OS of 39.5 months [20]. In the Giotag trial, all patients initially received 40 mg afatinib daily, followed by osimertinib if T790M acquired resistance was reported; the updated median OS was 37.6 months, while the OS was as long as 41.6 months in patients with an exon 19 deletion [30]. In the present multicenter retrospective study, the OS was similar in the 30 mg and 40 mg groups (34.0 and 25.2 months, respectively), suggesting that the clinical effectiveness of 30 mg afatinib daily as the initial treatment was not inferior to 40 mg daily as the starting dose for patients of stage IV lung adenocarcinoma with exon 19 or 21 mutation.

It should be noted that the current study enrolled patients from May 2014, and only 13 patients (13%) in the 30 mg group and 5 patients (6%) in the 40 mg group received osimertinib as the 2nd-line therapy. As osimertinib was very expensive and had not been reimbursed by the Taiwanese National Health Insurance until April 2020, the majority of patients with acquired-resistance to afatinib chose to receive platinum-based chemotherapy, rather than osimertinib, as their 2nd-line therapy.

The current study found that the predicting factors for poorer PFS might include more metastatic sites, no dose reduction, exon 21, pleural metastasis, and bone metastasis. The predictive factors for poorer OS might include male sex, poorer initial ECOG PS, more metastatic sites, smoking history, bone metastasis, and no adrenal metastasis. Liang et al. suggested that patients with significant pretreatment weight loss (> 10.0% in 6 months) had a shorter median PFS, and patients with brain metastases had a poorer ECOG PS status and were associated with a shorter median PFS [31]. Tanaka et al. also showed that patients with dose reduction had a significantly longer PFS than those without dose reduction in a real-world study (18.5 vs. 7.9 months, respectively; *p* = 0.018) [20]. However, the average daily dose of < 20 mg afatinib had a significantly shorter PFS compared with the other higher dose group (*p* = 0.049) [32]. Another study of afatinib in Taiwan showed that OS was not affected by reductions in the afatinib dosage; they also indicated that brain metastases at diagnosis and treatment response to afatinib are two important prognostic factors for OS [33].

Previous clinical trials recommended that 40 mg afatinib daily should be the starting dose in patients whose lung cancer harbors EGFR mutations, however, this dosage was often accompanied by serious ADRs and up to 28 to 53.3% of patients required dose reduction in the LUX-Lung 3 and LUX-Lung 6 studies [14, 18]. In a real-world study in Japan, 48% of patients receiving standard 40 mg afatinib daily had to reduce the dosage and 23% of patients discontinued treatment due to ADRs [29].

In fact, many clinicians had found more severe ADRs in patients who received standard 40 mg afatinib daily than those who received a 1st generation EGFR TKI, such as gefitinib and erlotinib. There is an urgent need to find a reliable strategy for reducing ADRs associated with afatinib, whilst maintaining its clinical efficacy for the management of lung cancer. Therefore, in clinical practice many clinicians prescribe a lower starting dose of afatinib [22, 23] or perform dose modification [19, 21] in order to improve patient outcomes and adherence. Recently, a prospective phase 2 clinical trial, which enrolled 46 patients assessed the efficacy and safety of lower starting doses of afatinib followed by dose modification, according to its toxicity in patients with EGFR mutation-positive NSCLC. The study had a median PFS of 15.2 months (95% CI: 13.2–not estimable) and the 1-year OS rate was 95.6% (95% CI: 89.7–100%) [22].

In a non-interventional, observational study [21] of patients who started with 40 mg afatinib daily, 67.1% underwent dose reduction, 86.5% of which occurred in the first 6 months. Dose reductions were more common in the females, East Asian individuals, and those with a lower body weight [21]. A post-marketing, observational study of afatinib in Japan found that a lower starting dose of afatinib was more commonly prescribed to the females and patients with lower body weight [20]. A study by Imai et al. enrolled 40 patients with a median age of 77 years (range, 70–85 years old) and all of them received 30 mg afatinib as the starting dose; their RR and median PFS were similar to the present study and their ADRs were also acceptable [19].

Since severe ADRs may discontinue the use of afatinib or lead to dose reduction, one must pay close attention to the incidence and severity of ADRs during the treatment of lung adenocarcinoma harboring exon 19 or exon 21 mutations. Of patients who received 40 mg afatinib daily as their starting dose in the phase 3 LUX-Lung 3 and LUX-Lung 6 trials, 73.0 and 80.6%, respectively experienced grade 3 or higher treatment-related ADRs; the incidence of ADRs dropped to 11.9 and 20.5%, respectively after the dosage was reduced [13, 14, 18]. In a real-world study, grade 3 or higher ADRs occurred in 30.4% of patients [20]. The present study demonstrated that acne and/or skin rash, diarrhea, dry skin, and paronychia were common ADRs and significantly fewer events were observed in the 30 mg group than in the 40 mg group, similar to the findings of our previous study [23]. Furthermore, patients who received 30 mg afatinib daily had a significantly lower incidence of severe ADRs than those receiving 40 mg daily, in terms of diarrhea, and acne and/or rash. Fewer severe ADRs might be associated with better drug compliance and a better overall quality of life for the patients. Besides, there was no increased incidence of recurrent central nervous system metastasis in patients receiving 30 mg daily afatinib as the starting dose compared with those receiving 40 mg daily initially in the current study.

Our study still had some limitations. Firstly, although the study enrolled patients from three hospitals, the retrospective design of this study might make the results less reliable than other standard prospective clinical trials. Secondly, the number of cases enrolled in the study was relatively low for a retrospective study. However, this study enrolled the largest number of patients receiving 30 mg afatinib daily as the starting dose to date. Thirdly, patients with recurrent lung cancer were excluded from the current study. Fourthly, we only enrolled lung adenocarcinoma patients and excluded those with squamous cell carcinoma or other rare types of lung cancer. Almost 99% of residents in Taiwan are covered by the Taiwan National Health Insurance and only adenocarcinoma harboring susceptible EGFR mutations is reimbursed. To obtain a more homogenous patient cohorts for investigating the factors associated with PFS and OS, we included only those initially diagnosed with stage IV lung adenocarcinoma in the current study. Fifthly, only 13% of patients in the 30 mg group and 6% of patients in the 40 mg group received osimertinib as their 2nd-line therapy. Most patients might receive a platinum-based chemotherapy, rather than osimertinib, as a salvage therapy because osimertinib had not been reimbursed by the National Health Insurance in Taiwan until April 2020. Further study is warranted to understand the effect of different 2nd-ling therapy on OS in patients receiving 1st-line afatinib for their lung adenocarcinoma harboring EGFR mutation.

In conclusion, a lower starting dose (30 mg daily) of afatinib for patients of lung adenocarcinoma harboring susceptible EGFR mutations showed similar RR, PFS, and OS compared with those receiving a standard 40 mg daily as the initial dose of afatinib. The lower starting dose was associated with fewer ADRs, as well as fewer moderate and severe ADRs. A further large-scale prospective trial is urgently needed to confirm these findings.

## Data Availability

The datasets used and/or analyzed during the current study are available from the corresponding author on a reasonable request.

## Abbreviations

NSCLC: Non-small cell lung cancer
ORR: Objective response rate
OS: Overall survival
PFS: Progress free survival
HR: Hazard ratio
RR: Risk ratio
ADR: Adverse drug reaction
HR: Hazard ratios
Cis: Confidence intervals
EGFR: Epidermal growth factor receptor
TKI: Tyrosine kinase inhibitor
ARMS: Amplification refractory mutation specific
PCRs: Polymerase chain reactions
ECOG: Eastern Cooperative Oncology Group
PS: Performance status
TTF-1: Thyroid transcription factor-1
PDL-1: Programmed death-ligand 1
RECIST: Response Evaluation Criteria in Solid Tumors
